# Validation of candidate gene-based EST-SSR markers for sugar yield in sugarcane

**DOI:** 10.3389/fpls.2023.1273740

**Published:** 2023-10-27

**Authors:** S. Divakar, Ratnesh Kumar Jha, D. N. Kamat, Ashutosh Singh

**Affiliations:** ^1^ Department of AB&MB, CBSH, Dr. Rajendra Prasad Central Agricultural University (RPCAU), Samastipur, Bihar, India; ^2^ Centre for Advanced Studies on Climate Change, Dr. Rajendra Prasad Central Agricultural University (RPCAU), Samastipur, Bihar, India; ^3^ Sugarcane Research Institute, Dr. Rajendra Prasad Central Agricultural University (RPCAU), Samastipur, Bihar, India

**Keywords:** sugarcane, EST-SSR, candidate genes, sugar content, association mapping, marker-assisted selection

## Abstract

Sugarcane (*Saccharum* spp.) is a widely cultivated crop that fulfils approximately 75% of the sucrose demand worldwide. Owing to its polyploidy and complex genetic nature, it is difficult to identify and map genes related to complex traits, such as sucrose content. However, association mapping is one of the alternatives for identifying genes or markers for marker-assisted selection. In the present study, EST-SSR primers were obtained from *in silico* studies. The functionality of each primer was tested using Blast2Go software, and 30 EST-SSR primers related to sugar content were selected. These markers were validated using association analysis. A total of 70 F1 diverse genotypes for sugar content were phenotypes with two check lines. All parameters related to sugar content were recorded. The results showed a significant variation between the genotypes for sugar yield traits such as Brix value, purity, and sucrose content, etc. Correlation studies revealed that the Brix%, sucrose content, and sucrose recovery were significantly correlated. An association analysis was performed using mixed linear model to avoid false positive associations. The association analysis revealed that the SEM 407 marker was significantly associated with Brix% and sucrose content. The SEM 407 primers are putatively related to diphosphate-fructose-6-phosphate 1-phosphotransferase which is associated with Brix% and sucrose content. This functional marker can be used for marker-assisted selection for sugar yield traits in sugarcane that could accelerate the sugarcane breeding program.

## Introduction

Sugarcane (*Saccharum* spp.) belongs to the Poaceae/Gramineae family of the Andropogoneae tribe. *Saccharum* and its species were commercially used for sugar production owing to their high biomass and sucrose accumulation (Godshall and Legendre, 2003). The sugarcane genome is complex polyploid in nature, with *S. officinarum* having a basic chromosome number of *x* = 10 (2*n* = 80) and *S. spontaneum x* = *8* (2*n* = 40-128). Thus, there are two distinct chromosomes that coexist in modern cultivars (D'Hont et al., 1994; Zhang et al., 2018).

Sucrose is a commercial component of sugarcane, and the improvement of sugar recovery is the primary focus of any crop improvement program. The identification of genes or marker for sugar yield is an important strategy for the improvement of sugarcane. The mapping of genes is a promising tool for characterizing genetic architecture such as yield component traits, such as sucrose yield, cane yield, stalk diameter, stalk height, stalk number, and stalk weight, as well as resistance to diseases, pests, and abiotic stresses (Aitken et al., 2008; Welham et al., 2010; Singh et al., 2013; Gazaffi et al., 2014; Margarido et al., 2015; Balsalobre et al., 2016; Balsalobre et al., 2017; Yang et al., 2018). The complex polyploid and highly heterogeneous genetic nature of sugarcane association mapping could establish the QTL from linkage disequilibrium between the markers and the trait. Surveying a large number of genotypes in the existing germplasm of sugarcane can be helpful in finding associations between the markers and traits, using association mapping (Wei et al., 2006; Banerjee et al., 2015). To avoid spurious associations, the population structure and kinship of the association map population were employed to elucidate inferences (Lander and Schork, 2006). Validation of all those markers linked with QTL will have been identified by means of association mapping in a diverse population (Korir et al., 2013; Picañol et al., 2013; Ukoskit et al., 2019).

The expressed sequence tag (EST) database was used to identify the targeted SSR markers because ESTs are considered effective for the direct association with the trait of interest (Dudhe and Sarada, 2012). The interspecific transferability of expressed sequence tags derived from simple sequence repeats (EST-SSRs) and genomic SSRs is well established (Wen et al., 2010). EST-SSR primers are more beneficial than anonymous SSRs from untranslated regions (UTRs) or non-coding sequences, being frequently more transferrable between closely related genera (Pashley et al., 2006; Chapman et al., 2009). Due to the primer target sequences’ location in the expressed DNA regions, which are predicted to be reasonably well preserved, there is a higher likelihood that the marker will be transferable across species borders (Varshney et al., 2005). EST-SSRs appear to disclose comparable amounts of polymorphism compared to SSRs found in UTRs despite their potential to reflect selectively harmful frame-shift mutations in coding areas. This is most likely because these coding regions have evolved to contain tri-nucleotide repeats (Ellis and Burke, 2007). Since EST-SSRs are physically connected to expressed genes, they constitute potentially useful markers. EST-SSR markers have a greater average rate of transferability between species than genomic SSRs because they reflect the expressed regions of a genome (Gupta et al., 2003). EST-SSR markers have been effectively used in gene tagging, linkage map construction, and QTL mapping (Qiu et al., 2010). Varietal crop improvement in various crops has become more feasible with the establishment of EST-SSR markers (Qiu et al., 2010; Ukoskit et al., 2019). EST-SSRs are highly regarded as a tool for breeding practices, perhaps because of their direct association with the genes of interest. It is also used in the identification of candidate genes in breeding and conservation input and population genetics studies (Yu et al., 2011). A mapping population obtained from a cross between commercial cultivars indulges in the introduction of EST-SSR markers into sugarcane linkage mapping (Oliveira et al., 2007; Palhares et al., 2012). There are some published reports of association mapping in sugarcane for traits such as biotic stress, cane yield, and sugar content (Wei et al., 2006; Wei et al., 2010; Débibakas et al., 2014; Banerjee et al., 2015; Gouy et al., 2015; Singh et al., 2016; Barreto et al., 2019; Fickett et al., 2019; Ukoskit et al., 2019; Coutinho et al., 2022). There are only a few studies that have investigated the identification of markers or genes for sugar yield traits using interspecific crosses (Reffay et al., 2005; Ukoskit et al., 2019). However, an association analysis requires a large population size and numerous EST-SSR primers. Moreover, these limitations could be avoided by choosing a diverse population with candidate genes for sugar yield for the validation of markers using an association study.

Therefore, the present study was conducted to identify different candidate gene-based EST-SSR markers from *in silico* studies, and these markers were validated using the association analysis of a diverse collection of sugarcane genotypes.

## Materials and methods

### Plant material

A total of 70 F1 diverse sugarcane genotypes for sugar yield traits were obtained from 14 different crosses, and five genotypes were chosen from each cross. The parents of all crosses were developed by crossing of *Saccharum officinarum* and *Saccharum spontaneum*. A few genotypes (BO102GC, BO137GC, and BO139GC) were also developed by general crosses (GC) for more variability. Seventy genotypes with two check lines (CoP16437 and CoP2061) were used in this study (**Supplementary Table S2**). These parents had contrasting natures for sugar and fiber yields. Because of limited seeds, these 70 genotypes were planted in an augmented complete block designed in the year 2021 at the Pusa farm of Dr. Rajendra Prasad Central Agricultural University, Samastipur, India. All genotypes were grown in seven different blocks, and each block had 10 genotypes with two check lines. The check lines were planted randomly in each block. The plot size of each block was 5.4 m^2^. All standard agronomic practices were followed to raise the crops.

### Phenotypic data and field data analysis

The population of 70 F1 genotypes was a phenotype for sugar-related traits after harvesting all genotypes. Brix, polarization (pol), sugar yield, and purity were recorded from four random stalks taken from each plot.

(1) Brix value (%): Brix value was measured using a hand-held refractometer. One degree of Brix is equal to the presence of 1 g sucrose in 100 g of the solution, and 1°Brix = 1% Brix. The strength of the solution was measured as a percentage of its mass. The reading was recorded using a refractometer with a sharp needle pierced through the stalk, and the collected substance was placed on a refractometer glass. The reading was recorded by the angle of the refractive index.

(2) Sucrose (%): Sucrose percentage was calculated using the following formula (Mehareb and Abazied, 2017):


$$\text{Sucrose }(\%)=\frac{\text{Brix}\%\times \text{purity}\%}{100}$$

(3) Purity (%): The percentage purity of the juice was calculated using the following formula (Mehareb and Abazied, 2017):


$$\text{Purity}\;(\%)=\frac{\text{Mass of the pure substance}}{\text{Mass of the impure sample}}$$

where pure sample: sucrose (%) and impure sample: Brix (%).

(4) Sugar recovery (CCS %): It was calculated using the following formula (Mehareb and Abazied, 2017):


CCS%=[Sucrose% −{Brix%−Sucrose%} x 0.4] x 0.73

(5) Sugar yield (t/ha)

Sugar yield was calculated using the following formula (Mehareb and Abazied, 2017):


$$\text{Sugar yield}=\frac{\text{Cane yield }\times \text{CCS}\%}{100}$$

### Phenotypic data analysis

Pearson’s correlation coefficients (*r*) between traits were calculated using the SAS CORR procedure based on trait means. Traits that were distributed normally with the Shapiro–Wilk test were considered normal data (Weber and Moorthy, 1952). Morphological data were used for the principal component analysis (PCA) using R studio. The data were imported in Excel format, and the eigenvalue must be greater than 1 for the variables. A bi-plot analysis was conducted using eigenvalues.

### Extraction of DNA

A total of 500 mg of young leaf tissue was collected for marker analysis, and DNA was extracted using the cetyltrimethylammonium bromide method of Srivastava and Gupta (2008). DNA quantity and quality were determined using 1.0% agarose gel electrophoresis and nanodrop spectrophotometry, respectively.

### Identification of a suitable EST-SSR marker and its validation by association study

Sugarcane is a polyploid crop, and its genome size and structure vary from genotype to genotype. Therefore, EST-SSR markers are best suited for tagging complex traits such as sugar yield. A total of 213 EST-SSR primers of sugarcane were identified in an *in silico* study (**Supplementary Table S1**). Furthermore, the functionality of these primers was tested using Blast2Go software. A total of 30 EST-SSR primers related to sugar yield were selected for this study based on their functionality (**Supplementary Table S3**). The PCR products were separated at 3.5–4.0% agarose gel. Out of 30, a total of 25 primers were amplified by PCR and used for further analysis. All 25 primers were scored based on the presence (1) or absence (0) of bands in the 70 genotypes of the mapping population.

### Validation of EST-SSR primers using association mapping

The similarity coefficient among the genotypes was calculated using the genetic distance (Nei and Li, 1979). A neighbor-joining dendrogram was constructed using Past3 software. N-J analysis was performed using multivariate clustering, and the tree was constructed by Euclidean genetic distance with bootstrap replications of 100. The population structure was analyzed using STRUCTURE software to estimate the number of groups/subpopulations by setting the burning period length to 100,000, and each value of *K* was run three times with the *K* value varying from 1 to 10. Furthermore, a *Q* value below 0.9 was described as an admixture. An association analysis was performed using a mixed linear model as described by Yu and Buckler (2006). It was performed using TASSEL incorporating the *Q* matrix and *K* matrix to avoid false positives. The significant threshold for association was set at *P<*0.05.

## Results and discussion

Sugarcane is polyploid crop with a complex genome, and it is difficult to interpret genome data. Less information about the sugarcane genome makes gene manipulation very difficult, but the identification of the functional genes responsible for sucrose accumulation makes it possible. EST-SSR markers were considered to be a highly regarded breeding tool as they are able to localize the functional gene by marker association (Palhares et al., 2012) since they may be directly associated with the gene expressing a particular trait.

### Phenotypic yield data analysis

The germplasms used in this study were produced using an inter-varietal cross to validate primers for sugar yield traits in sugarcane. The yield distribution showed that the selected genotypes varied for sugar yield traits (**Supplementary Figure S1**). These germplasms were derived by crossing the sugarcane genotypes with contrasting sugar yield, and a few crosses were produced by general cross. The maximum, minimum, and mean values of all five phenotypic traits showed variation in the population for sugar yield traits. The Brix (%) values vary from 19.27% to 22.72%, with a mean of 20.65%. Similar trends were recorded for purity, sucrose content, sugar recovery, and sugar yield. This indicates that the selected germplasm shows variability and is appropriate for the study. The phenotypic correlations between the five traits were significant. The highest phenotypic correlation was found between sucrose content and sugar recovery, while the lowest phenotypic correlation was found between Brix value and purity (**Table 1**).

**Table 1:** Mean performance and the correlations between traits at the time of harvesting of sugarcane.

Statistics	Brix	Purity	Sucrose content	Sugar recovery	Sugar yield
Mean	20.65	85.88	18.12	14.33	10.4
Maximum	22.72	87.79	20.06	15.97	16.41
Minimum	19.27	83.45	16.9	13.2	6.76
Correlation^a^					
Brix					
Purity	0.127^ns^				
Sucrose content	0.952*	0.139^ns^			
Sugar recovery	0.947*	0.208*	0.981*		
Sugar yield	0.272*	0.345*	0.267*	0.301*	
Significant* *P<0.05*					

### Population structure and genetic relationship

**Figure 1:**
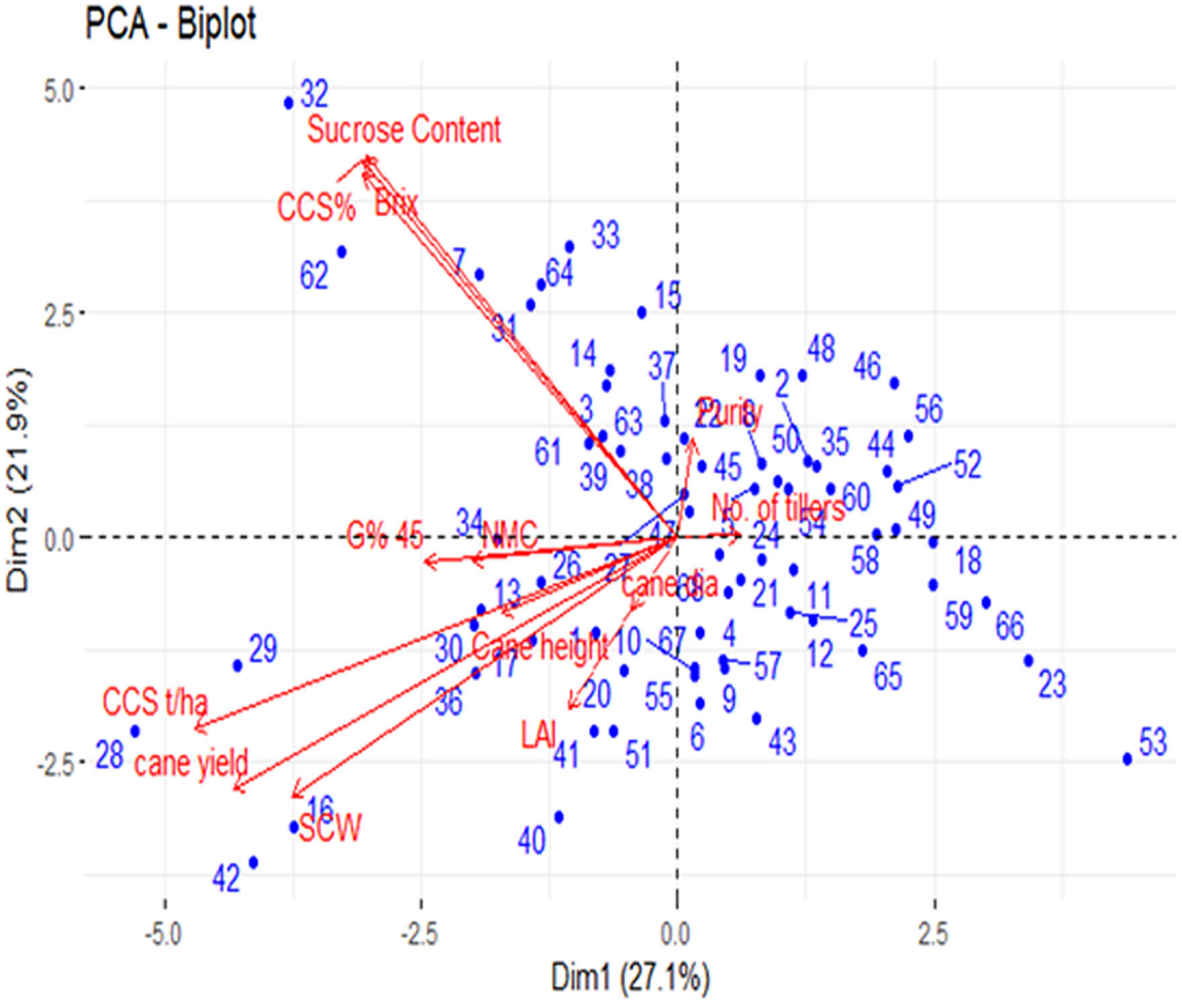
Biplot of principal component analysis PC1 (Dim1) and PC2 (Dim2); Where the dot represents the genotypes (70); Arrow represents the traits.

Many studies were conducted on sugarcane considering its complex polyploid genome, and several assumptions are not fulfilled for its complex structure; therefore, the applicability of this algorithm may be limited in sugarcane (Wei et al., 2006). Analyzing the sugarcane subpopulation using Structure software and mixed linear model provides an opportunity to track the gene related to complex traits of sugar yield in sugarcane. In the present study, minimum population size with a maximum variation was used, which is the most favorable for association analysis (Wei et al., 2006). Spurious associations were controlled, while the power to detect true associations was maximized using PCA as a random component to control for population structure (Pastina et al., 2012). PCA, as a random component, is included in the analysis, and the large population structure is captured with the first few axes that account for most of the variation, while the more subtle relationships among individuals are captured by the remaining significant axes. The population showed a clear and continuous variation in its structure, PCA, and neighbor-joining dendrogram (Fickett et al., 2019; Ukoskit et al., 2019). Furthermore, most of the structures found in these genotypes seem to originate from subtle kinship relationships rather than a large-scale population structure. The mapping population was diverse and highly heterozygous, and environmental conditions play a major role in the formation of sugar. The PCA results showed that Brix%, sucrose content, and CCS% were highly positively correlated, and the CCS and cane yield are highly negatively correlated (**Figure 1**). The results of the PCA and neighbor-joining dendrogram were coherent and showed no disjuncture in the population. The biplot of PCA overlays both individuals and the variables in a single graph. The loading range was varied from -5 to 5. The high absolute loadings were directed to either positively or negatively describe the variable that strongly influences the component, and a value less than that of the high loading indicates that they had a weak influence on the component. Sucrose content, Brix, and CCS% were highly positively correlated, as identified by PCA, and they showed 49% of the total variation in the phenotypic data (**Figure 1**). Hence, the traits directly related with sugar yield showed significant variability and indicate the diverse genotypic nature of germplasms.

The markers were identified by surveying the sugarcane database SUCEST, with their functionality scored by BLAST to determine the homology and putative function of the marker sequence (**Supplementary Table S1**). EST-SSR markers are derived from the expression regions of the genome and have greater potential for the direct association of the trait. The data Blast2Go showed that, at every 18.60 kb, one SSR motif was found to be very similar to cotton and wheat (Cardle et al., 2000). Pinto et al. (2004) reported the density of SSR in sugarcane at every 16.90 kb.

**Figure 2:**
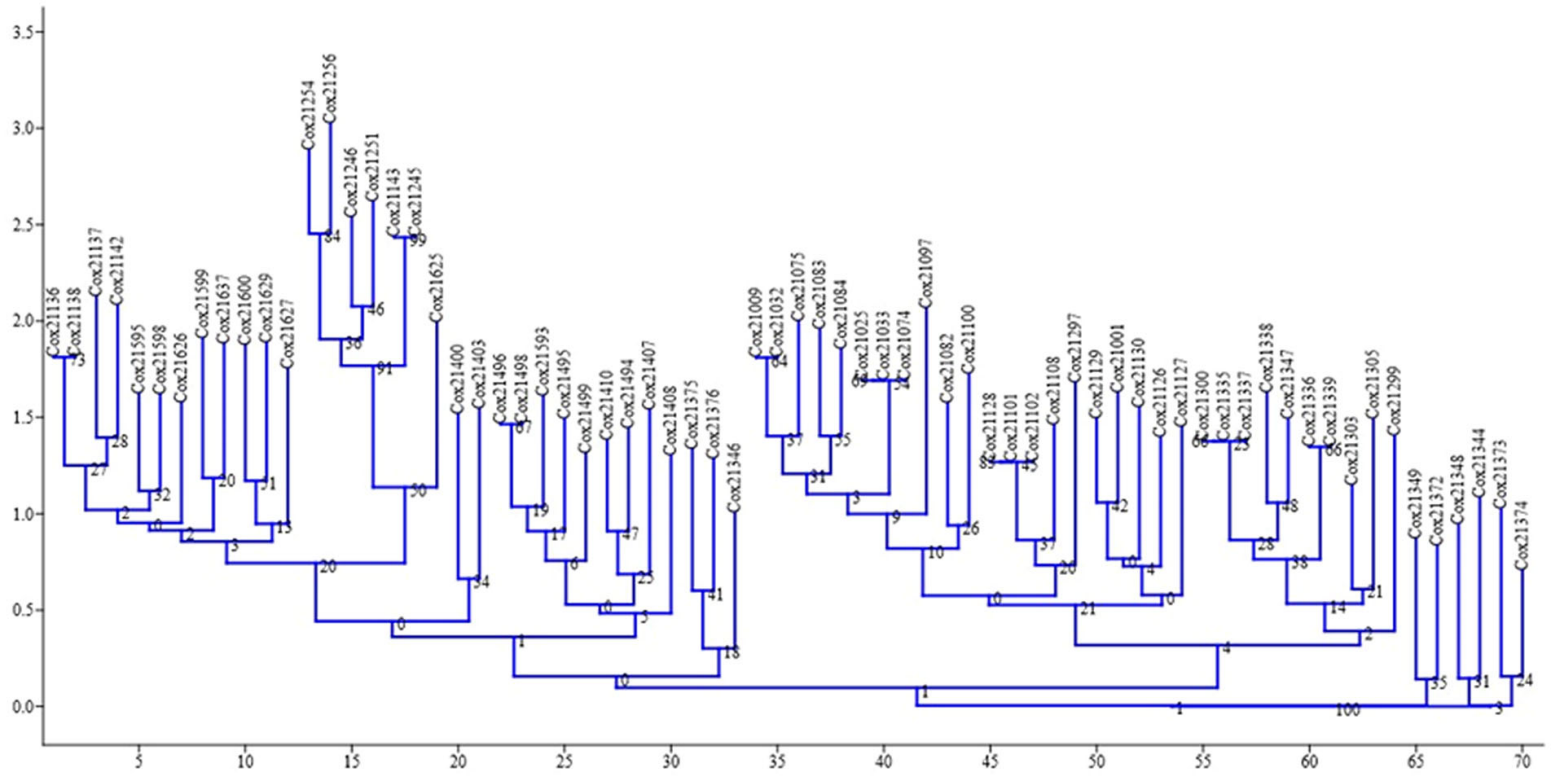
Dendrogram showing the relationship among 70 genotypes of sugarcane using EST-SSR markers.

The neighbor-joining dendrogram is a bottom-up clustering method designed to provide a single tree and may be able to produce more than one dendrogram from the same data (Saitou and Nei, 1987). This method provides faster and better results than UPGMA, and most implementations provide a single tree (Page and Holmes, 2009). The dendrogram of the neighbor-joining relationship based on EST-SSR allele frequencies separated the populations into three differentiated clusters: A, B, and C. Cluster A has 33 genotypes, cluster B has 31 genotypes, and cluster C has six genotypes (**Figure 2**).

**Figure 3:**
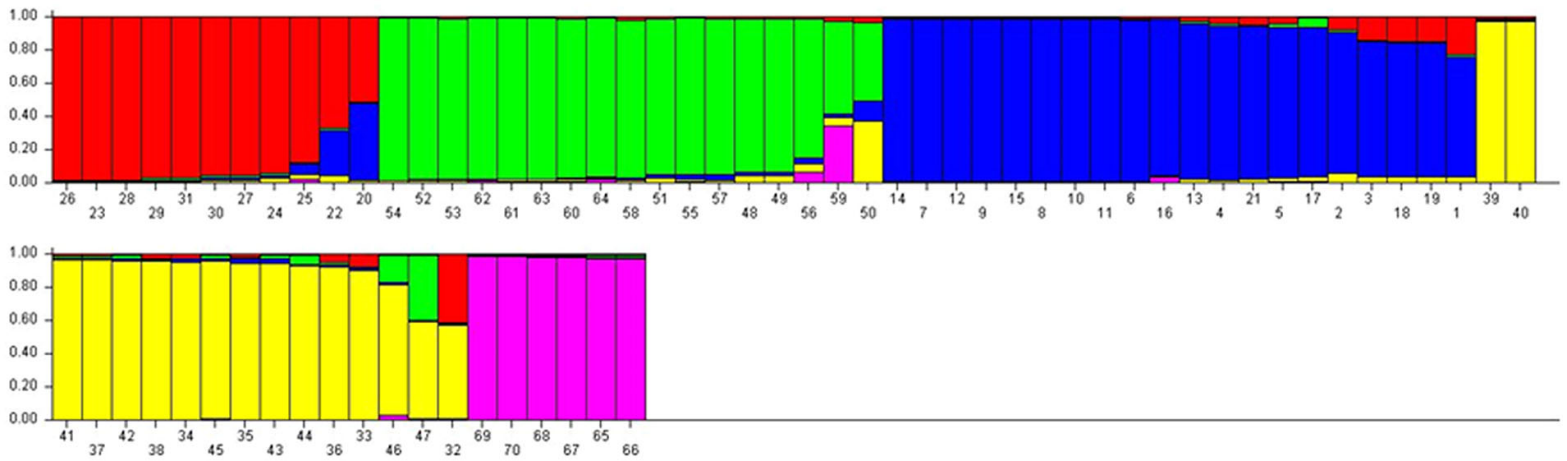
STRUCTURE analysis of bar plot. Populations with one solid color that is not shared by another group are genetically distinct Populations that share colors are more similar. Bar graphs for five sub-populations are indicated by different colors. The vertical coordinates indicate the membership coefficient of each individual and the horizontal represents the genotypes.

**Figure 4:**
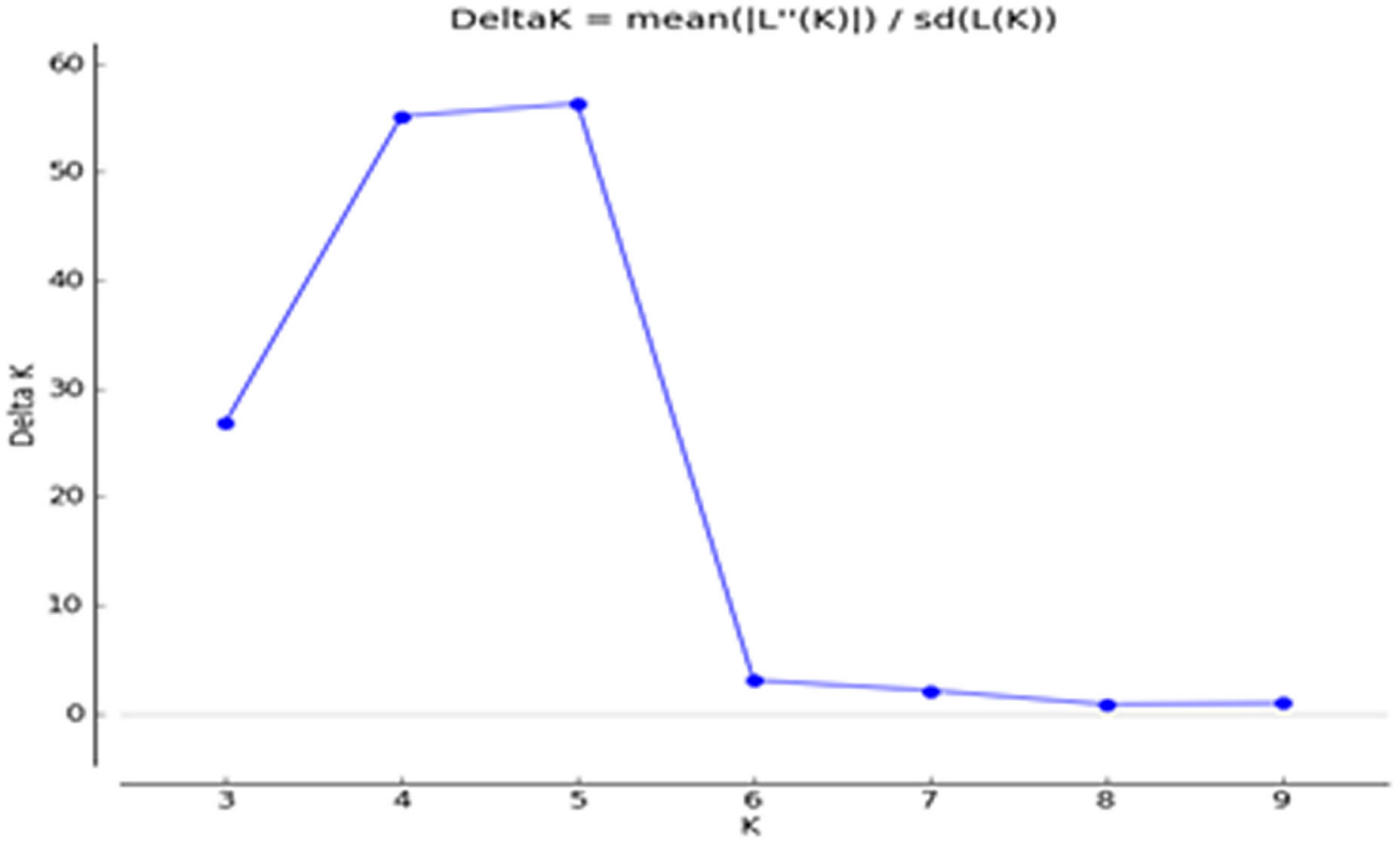
Delta K of STRUCTURE software using Evanno’s criterion.

The Q matrix was calculated, and grouping of the population was performed using genotypic data. The result showed the marker-based kinship of the population. The number of subpopulations (Δ*K*) was identified by structure and maximized at *K* = 5. Populations with a single color were not shared by any other group, indicating that they were genetically distinct. The populations showed that the sharing of colors was similar to that of another group of individuals in the population (**Figures 3**, **4**). The marker-based kinship matrix underlying the study of Yu et al. (2006) was determined based on the definition that random pairs of genotypes are unrelated, whereas Zhao et al. (2007) defined pairs of genotypes that do not share any allele as unrelated. The results clearly indicated that most of the genotypes were different.

### Validation of EST-SSR markers

**Figure 5:**
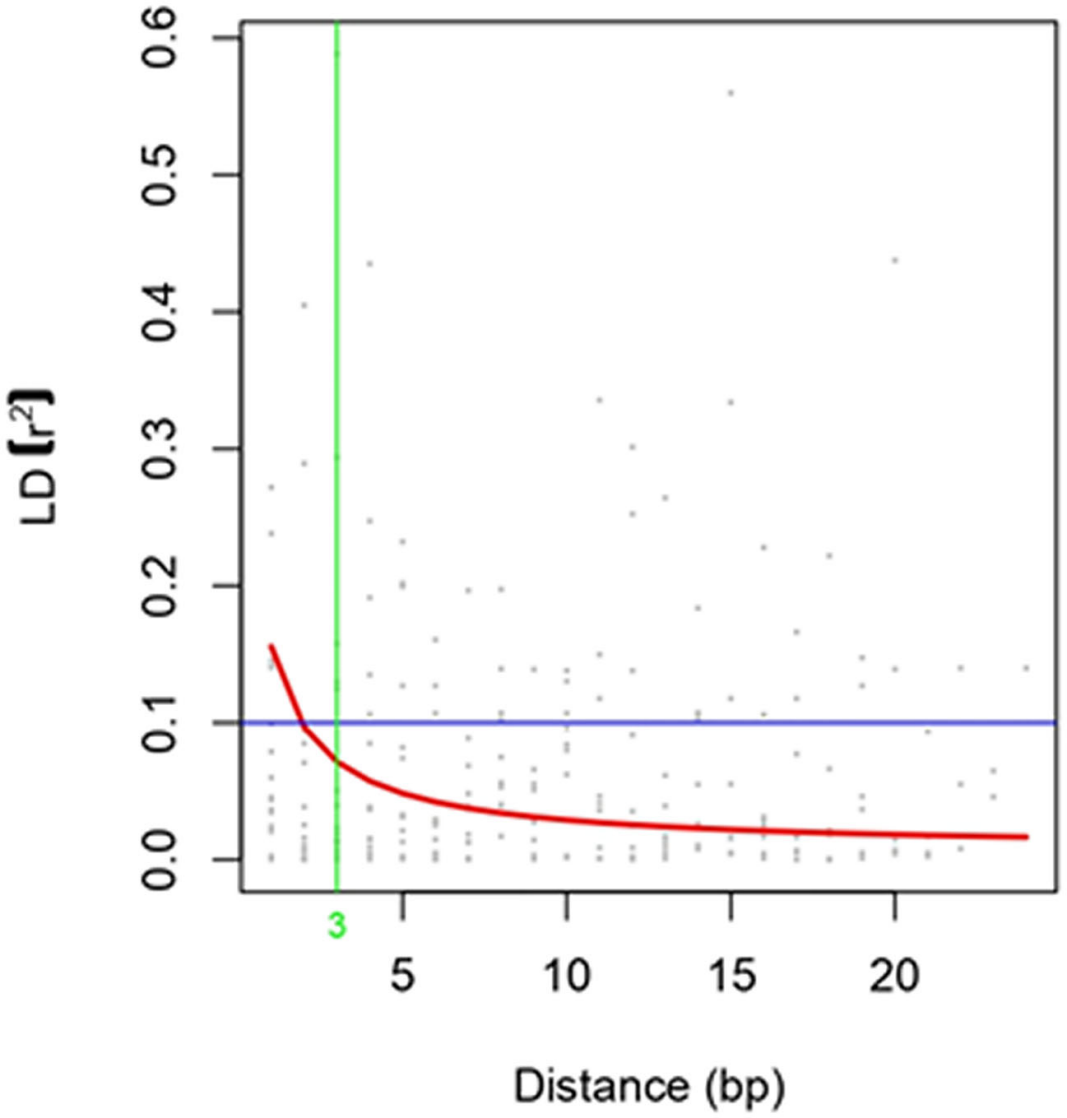
Linkage Disequilibrium Decay Plot.

Sugarcane crops are polyploid and exhibit a high level of variation in the F1 generation. Therefore, validation of primers using a simple chi-square is not possible. Hence, the primers were validated using association mapping. If the number of primers is a major limitation of the study, then the candidate gene approach for association mapping is best suited. A total of 30 EST-SSR markers were used for the association analysis of sugar yield traits. Of the 30 primers, 25 were amplified, and all these primers were tested for association studies. EST-SSR markers are a tool for association studies (Ukoskit et al., 2019; Coutinho et al., 2022). The EST-SSR markers related to sucrose content could be more effective than markers that focus on the varied functions of the gene. The linkage disequilibrium (LD) decay plot for the *r* ^2^ values between the markers was plotted against the genetic distance. The highest frequency of loci pair in LD is mapped less than 3 bp. The lowest frequency of loci pair was more than 20 bp, indicating that the probability of LD is low between distinct loci pairs. The majority of the loci pairs in LD with *r* ^2^ >0.01 at *P<*0.05 were found in ≤ 20bp (**Figure 5**). The values decreased as the genetic distance between the loci pairs increased. EST-SSR marker represented by the different regions of the genome was associated with the trait of interest at a *P*-value of 0.05. SEM407 was significantly associated with Brix%, sucrose content, and sugar recovery (CCS%) (**Table 2**). Except for SEM407, other EST-SSR markers did not show any association with sugar yield traits because the functional allele discovered in the mapping population might not be recognized in plants because it is rare in the larger germplasm. Compared to the genes tagged in the mapping population, the sugarcane accessions in the association population may have various trait-related alleles of various genes at various sites. Additional functional alleles that are absent from the mapping population may be found by validating the marker–trait relationship (Peace and Norelli, 2009). Although the number of markers used in this study is relatively low, the marker–trait association of SEM407 was significant. This marker was found to be significant for the sugar-related yield traits of genotypes. These results suggest that the association approach used in this study is consistent with the detection of QTL associated with sugar yield traits. The marker identified to the respective QTLs or genes should be used for marker-assisted selection (MAS). MAS for simply inherited traits are gaining increasing importance in breeding programs, allowing the acceleration of the breeding process of sugarcane (Francia et al., 2005). This study would be helpful for the plant breeders in marker-assisted selection in the prospect of achieving higher sugar yields while designing their crossing program.

**Table 2:** Associations study between EST-SSRs and sugar-related traits at P<0.05.

Markers	Association mappin2				
	Brix%	Purity	Sucrose content	Sugar recovery (CCS %)	Sugar yield
	P value	P value	P value	P value	P value
**SEM2**	0.07	0.47	0.08	0.1	0.2
**SEM58**	0.29	0.44	0.3	0.3	0.38
**SEM112**	0.3	0..2	0.29	0.3	0.2
**SEM117**	0.70	0.68	0.5	0.47	0.25
**SEM159**	0.70	0.77	0.64	0.62	0.22
**SEM168**	0.72	0.39	0.79	0.82	0.74
**SEM191**	0.99	0.26	0.84	0.90	0.56
**SEM199**	–	–	–	–	–
**SEM203**	0.43	0.91	0.41	0.43	0.96
**SEM358**	0.89	0.5	0.77	0.82	0.39
**SEM368**	0.12	0.85	0.3	0.27	0.48
**SEM369**	0.95	0.86	0.59	0.57	0.76
**SEM407**	0.002*	0.70	0.002*	0.002*	0.70
**SEM425**	0.29	0.40	0.2	0.1	0.55
**SEM428**	0.92	0.77	0.95	0.95	0.97
**SEM430**	0.58	0.61	0.56	0.58	0.38
**SEM432**	0.91	0.25	0.98	0.93	0.92
**SEM433**	0.63	0.46	0.7	0.63	0.56
**SEM435**	0.83	0.1	0.77	0.86	0.57
**SEM436**	0.73	0.87	0.62	0.63	0.41
**SEM437**	0.15	0.86	0.12	0.13	0.28
**SEM439**	0.76	0.12	0.74	0.80	0.11
**SEM440**	0.92	0.43	0.98	0.93	0.43
**SEM454**	0.46	0.96	0.59	0.56	0.30
**SEM456**	0.99	0.79	0.87	0.85	0.23

The experiment-wise threshold was based on the Bonferroni corrected method.

The thresholds for marker significance were at *P<0.05.

## Data availability statement

The original contributions presented in the study are included in the article/**Supplementary Material**, further inquiries can be directed to the corresponding author/s.

## Author contributions

SD: Writing – original draft, Data curation, Formal Analysis, Methodology. RJ: Writing – review & editing, Funding acquisition, Supervision. DK: Writing – review & editing, Data curation. AS: Writing – original draft, Conceptualization, Funding acquisition, Supervision.
