# Supplementary material for: Validation of candidate gene-based EST-SSR markers for sugar yield in sugarcane
Source: Front Plant Sci. 2023 Oct 27;14:1273740. doi: 10.3389/fpls.2023.1273740 (PMC10641762; doi:10.3389/fpls.2023.1273740)
Supplement: Supplementary file 1 [file DataSheet_1.docx]

| **Supplementary Table 1:** List of EST-SSR primers identified from the sugarcane database related to different traits | | | | |
| --- | --- | --- | --- | --- |
| **S.No** | **PRIMERS NAME** | **FORWARD PRIMER** | **REVERSE PRIMER** | **FUNCTION** |
| 1 | SEM1 | ACCATCGCAATCGATGTTTA | AACTGGATGGCGTACAATCA | hypothetical protein |
| 2 | SEM2 | ACACCGAGCTGTCCCAAT | GCATCTGATGAGCCTGTGAA | glycosyl hydrolase family protein 17 |
| 3 | SEM7 | GGAAGGTATGGGTGCTATGC | ACAGGGCAATAACAGGGGTA | Hit not found |
| 4 | SEM11 | AGGGCTTGGAAGAAAGGAAT | TGGCAAGCAACAGCTAAAAC | hypothetical protein OsJ_018777 |
| 5 | SEM13 | GCGAGAGAAGCTAGGAAGCA | CGCAGATCCTCTTGAACCTC | lysine decarboxylase-like protein |
| 6 | SEM14 | GAGCAACGAGCTGAAAAGTG | TGTCCTGACCTAGGATGTGC | Hit not found |
| 7 | SEM15 | TGTCCACAATTTTGGCTGAT | GTTGCTTGCCTGATCATTGT | Hit not found |
| 8 | SEM16 | CCTTCCTTGGCCTCTTCTCT | TGCTGGTCGCAGTACTTGAT | harpin-induced protein 1 family (HIN1)-like |
| 9 | SEM19 | CAGCCCATTAACCAAGCAAT | GAAGCAGCTGTTGCTCACTG | ARFE_ORYSJAuxin response factor 5 |
| 10 | SEM20 | CCGGCTGTGAAAATTAGGTT | TCGAATTGGTCAAGACTCTCC | NADH-plastoquinone oxidoreductase subunit K |
| 11 | SEM24 | CAATTCGTGGCTTGTGTTTG | AGCAGAATCGGCAAGGTAAA | Hit not found |
| 12 | SEM27 | AGGTCTCGGCACTTGGAGTA | GATCGATCCTCCCTCTTTCC | mediator complex subunit SOH1 |
| 13 | SEM29 | GATCGATCCTCCCTCTTTCC | AGGTCTCGGCACTTGGAGTA | Probable mediator complex subunit SOH1 |
| 14 | SEM36 | CCATGTGCAGCATTTAACAA | TGGACATGCTAATGACTACTGC | Hit not found |
| 15 | SEM37 | CTTCCTGCTTCGAACATTTG | ACGAGGTAGATCCCGAAGG | unknown protein |
| 16 | SEM38 | TGAATTGCACAAAACACCAA | GACGGTGTAAACAAGCTGTGA | WD-repeat containing protein |
| 17 | SEM41 | CCCCCTTGACACCTCTGTAT | TAGAACGAACCAGACGACCA | Hit not found |
| 18 | SEM42 | GACTTCGGGAAGAAGGAGGT | ACCAAGCACATCCAGCAGTA | hypothetical protein |
| 19 | SEM46 | GCGGTCTTGTAGTGGTAGAGC | TGATCCTCCTGTTCCTCACA | pi starvation-induced protein |
| 20 | SEM53 | CCGCCTTCTCCTTAGTGACA | CCACAAGCCTAATACAGCTCAA | hypothetical protein OsJ_026388 |
| 21 | SEM55 | ACGGCATCAGATTCAGATCA | ATGGCTTTCCATCTCGTGAC | zinc transporter |
| 22 | SEM57 | TCCAGAAGTACGTGGAGACG | ACGACAGCAGGTCGAACAT | Hit not found |
| 23 | SEM58 | CCAACCAACCTCGACATTCT | CCATGTGATCTGACCTGGTG | sugar-starvation induced protein |
| 24 | SEM60 | TGCTAACACATTTCAAGAAAGAGA | GATCCAATCCGAGGAAAAGT | unfertilized embryo sac 16 |
| 25 | SEM61 | GTTCAGAACACGTGCAGCAT | CACGCTTGACATGAGAGGAA | zinc-finger protein |
| 26 | SEM63 | GGTCGGTGCTCTGTTCTTTT | CCTGCAGCAGAGACGAGAT | Hit not found |
| 27 | SEM68 | CCCTGAGGTCTCTCTCCACT | TGCCATAGGACAAGAGTTTAACA | Hit not found |
| 28 | SEM72 | GAACCTTATCGGTAGCCTCCT | GAGCGCCATAGGAGAAGTG | 30S ribosomal protein S17, chloroplast precursor |
| 29 | SEM76 | ATATGCCATGCACGTGTGAC | CAATGTAACCATCGCAGCAT | cysteine proteinase |
| 30 | SEM78 | GTGGTCGCAGACGAGGTC | CTCCGCATTAGCCATTTCC | ubiquitin-protein ligase-like |
| 31 | SEM79 | GATGGAACAGATGCGACAAG | GTTCATCGTAACCTGCTGGA | Nitrilase-associated protein |
| 32 | SEM80 | GCAGATGAGAGGGCAAAAGT | CGCCTGCAGATGAATCATAG | heavy meromyosin-like |
| 33 | SEM82 | AGTACAAGGCACAGCCAGAG | GGACATGAGGTACACCCAGA | cbs domain-containing |
| 34 | SEM83 | TCCTCCTCTTGTTGCAGTTG | GTCGTCGTCACGATCATCTC | Dof zinc finger protein MNB1A |
| 35 | SEM84 | TGTAGCAATTCCTTGCGTTG | CAACAAATACAATGCCAATCG | Hit not found |
| 36 | SEM85 | CACCTAGTGAAAGGGGCAAA | CCTGAAGCCTTGGTAGCATC | pectin-glucuronyltransferase |
| 37 | SEM86 | CGAGAACTAGCATAGCACAAGA | AACAACTGGTGCAAGTCCAT | chitin-inducible gibberellin-responsive protein |
| 38 | SEM90 | CGTGGGCTAATACAGCTCAA | CCCACTGGCACAATCTTCTT | hypothetical protein |
| 39 | SEM92 | TGTGCCGTTGCCTAATAACA | GGCAAGCTTCCTCAGTTTCTT | bZIP transcription factor |
| 40 | SEM94 | TTATCTCTTCCCTGCGGTCT | CTGGCTGGGGAGCAACTT | Hit not found |
| 41 | SEM97 | TTTGGGGGTTCTTAGTCCAG | GAAGACAGTGGACGAGGTCA | ankyrin-like protein |
| 42 | SEM98 | GCCAGCAGAATGCTTAACAA | TCTTACTCTGTCCCCCAACC | Hit not found |
| 43 | SEM99 | TTACCTCCGGCAACGTTAAA | TGCAGGCATATGGTAGTCCA | Hit not found |
| 44 | SEM105 | AGGGGCCTCAAGTTGTTCTT | CGGTCTCATGGTCACCTTTT | exocyst subunit EXO70 family protein |
| 45 | SEM106 | AGGTTGCTGATGGTCCTCAC | CAAGAAGGGAAGCAGGGACT | hypothetical protein |
| 46 | SEM108 | CGACTTGTGTGGAGGTGAAA | TGGATCAATGTGAACAAAATCG | VP15 |
| 47 | SEM110 | GTCTCCTCCTCAACCACTGC | GTGGGGTATGTAACCCATGC | Cortical cell delineating protein precursor |
| 48 | SEM112 | CCTTCTGCAGACGAGTTGAA | ACCTGACCAGCAAATCAACA | S-adenosylmethionine decarboxylase |
| 49 | SEM113 | TTCCGGTTTACCCTGCATAG | TTCCTCAGGGCCCTTTTATT | hypothetical protein |
| 50 | SEM117 | GCGTGGCACTGACTACAAGT | CAATGTTCTGTGGTCTGCACT | nuclease I |
| 51 | SEM119 | CCGCGTGCTCTCTCTCTCT | ATTGCCATCACCTCATGCTT | hypothetical protein OsI_018669 |
| 52 | SEM120 | TAGCAGCTCGATTCACGATT | ATCCTAGTGTGGTGGGTGCT | protein |
| 53 | SEM124 | CCTTGATGTGCTTGACGAG | CCAACGAGCAAAAGTAAACG | bet v i allergen-like |
| 54 | SEM125 | TATTCTCTCCGGATCCCCTA | ATTCAAAGCGCAACACAGTC | hypothetical protein |
| 55 | SEM127 | GCAGGATAAGCTTGGCAGTT | CTCAAGGCAAGGAACGAATC | Hit not found |
| 56 | SSEM131 | AAAGGAAAGCAAACCCAAGG | GCAGTCGTTGTCGTAGCAGA | lipid transfer protein precursor |
| 57 | SEM132 | CCCTCCACCTCTTTGCTC | TAGAAAGACCTGCCCTCCTG | RRM-containing protein |
| 58 | SEM136 | CTCTGACCCGAGCAAAGG | GCCAATAAACAGCAGGGGTA | Hit not found |
| 59 | SEM140 | GTGTTTTGGAGACCGTGTCA | CGATTGTTGCGCTGTACATC | Hit not found |
| 60 | SEM141 | ATCATCCACAGCTAGCAGCA | GGTTTTGCCTTGGTTTTTGA | GRC14_ORYSJPutative glutaredoxin-C14 precurso |
| 61 | SEM143 | CCCCTCTCCCTCAGTCTTCT | CCATGCTGTCAGGATCCAC | Hit not found |
| 62 | SEM147 | GCCTCTTCCTCCTCCACTC | GACGACCGTCCTTGTTGAG | hypothetical protein OsJ_003463 |
| 63 | SEM148 | GCTACCGGATGGATAAAAGC | CTGACCGAAATGATCAAGGA | Hit not found |
| 64 | SEM154 | ACCGAGGTAGGAGGGAGTGT | GCTCGCCATGAATAGAAAGG | ribosomal protein L4/L1 family protein |
| 65 | SEM158 | GGATGGTTAAAGCGGAAACA | GGAAACAGTGTACGCCCAGT | Hit not found |
| 66 | SEM159 | CTGGTGGAATAACTCGCTGA | CTCAAGGCAAGAACGAATCC | exhydrolase II |
| 67 | SEM161 | GAACTGCTCACTGGCTCCTC | GTAGAAGTCCGTCGCCGTAA | auxin efflux carrier |
| 68 | SEM164 | GAGGTTATGGGGAAACCAGA | GATTGCAGCCGTAAACTTGA | Hit not found |
| 69 | SEM166 | AGCGCATCTTGCTTATTTGA | ATGCATGATCATCGAGGAAG | Hit not found |
| 70 | SEM167 | CGGATCTTGGCTCCTTCTCT | AGCCTTGATTGGCAATGGTA | Hit not found |
| 71 | SEM168 | AACGTCAGCCGCTACAACTT | CTTCCCTTTTGCGAAGAAAA | glucanase |
| 72 | SEM174 | CTCACCGCAGCTCTTTTTCT | CACAAGCTATGCGGTCAAAA | hypothetical protein |
| 73 | SEM176 | CGCCATAACCATAACCACAG | CCTCCCTCCGCTACTTCCTA | (DSRNA-BINDING PROTEIN 3); double-stranded RNA binding |
| 74 | SEM179 | TATTCCACCGGGAACAAGAA | GGGATTGTAGCGACGAGTTG | receptor protein kinase PERK1 |
| 75 | SEM180 | TTCCACATCAAGCAAGCAAG | ATGACATCAGGAGGGAGACC | hypothetical protein OsI_012598 |
| 76 | SEM184 | ACCAACGCGACGAGAGAG | GCCTGAACTGGTCGTAGGTC | positive transcription elongation factor/ zinc ion binding |
| 77 | SEM189 | GAACTGCTCACTGGCTCCTC | GTAGAAGTCCGTCGCCGTAA | auxin efflux carrier |
| 78 | SEM190 | CTAGCACGGCAATACAGGC | AGATCTGTTGGGTGCTCGTC | Hit not found |
| 79 | SEM191 | CCAGTCGCGATTCTTCCAC | AAGGGACGGGGAGAAAAATA | Hit not found |
| 80 | SEM195 | CTTCCCGTCGCTCTTACCT | CTCCTCCTCCTCCTCTCCAC | Hit not found |
| 81 | SEM199 | CTCTCGAGGAGGTGGATGAG | CTGCAAGTTTGTTGGCTGAA | hypothetical protein OsJ_007772 |
| 82 | SEM200 | CTGCAGGATCACCTGGAAC | TAAACCCACGCTGACAGACA | cytochrome p450 |
| 83 | SEM203 | GCGGCCTCATACGTGTAGAT | TCTCTTCCCCTCACCAGAAA | wd40 repeat protein |
| 84 | SEM206 | CATGGTAGCTCCGCTTCTTC | GCGAGAAGCTAGGAAGCACA | lysine decarboxylase-like protein |
| 85 | SEM207 | GGCACACCTCGAGAGACC | ACTCCTCCTCCTCGCTTAGG | disease resistance protein |
| 86 | SEM208 | TGAAGACGATGATGGGATGA | TCTGTTTTGCTCCTCCGTCT | phytoene synthase |
| 87 | SEM211 | CGGTCGTCTCTTCCTCCTC | CTACTACCACCCGGACCAGA | hypothetical protein OsI_020099 |
| 88 | SEM213 | CTCTCCGACTCGTCTTCCAC | GCGGACTGCAAAAGAGAGAT | Hit not found |
| 89 | SEM214 | ATCGGCTCCAGTCAGAGAGA | CCTGGTGAAGGCTCATGATT | Hit not found |
| 90 | SEM215 | GCCGAAGAGGAATCTACGAG | GTTTGTCTTCCTCCCTGTGC | Hit not found |
| 91 | SEM217 | CACGGGGAGACGAGAGAC | CCAACAACAACCAGAATATCG | hypothetical protein |
| 92 | SEM219 | AAGTACGGAGCGCAGTGTAG | ACCGCCTTGTACTCCAAATC | zinc finger |
| 93 | SEM220 | AAGCTCCTTGCCTGCTACTC | CAAAGGGCATCCTTTCTGAT | Chitin-inducible gibberellin-responsive protein 1 |
| 94 | SEM221 | GCCTCTCTCTGCTCAGCCTA | CTCCTCATCTCTCGCCAAA | calcium-responsive transcription coactivator |
| 95 | SEM223 | CACAGCACTTGCCAAGCTAA | AGTTTCACAAAGGGCGACTG | DNA-dependent RNA polymerase II |
| 96 | SEM227 | AGTTTCACAAAGGGCGACTG | CTAGAAGCAGAAGTGGAGTGCT | GT-2 factor |
| 97 | SEM229 | AGAACCACAACCACCAGGAG | ACAGTTGAATAGGCCGGATG | diphosphate-fructose-6-phosphate 1-phosphotransferase |
| 98 | SEM231 | CCGTTCTACACCTCCAACAT | GACCGTGACCATCTGCTG | lateral root primordia |
| 99 | SEM232 | CAACTCCAGCTCCAGTCTCC | CTTTTCGCGAAGTGAACACA | ubiquitin C-terminal hydrolase |
| 100 | SEM233 | TTGCTTGGGACAAAAGGCTA | ATCTTGCAAAGGAAGGAGCA | gene X-like protein |
| 101 | SEM234 | GGACATGCTGCTCCCTACAT | AGGAGGACTGGTGGTTGAGG | family transcription factor containing protein |
| 102 | SEM235 | CATCGGCTCATCATAACGAA | AGCTACTTCAGCCCCAAGTG | Auxin-responsive GH3-like protein 1 |
| 103 | SEM236 | CCCTTTGCTTCCCCTTTACT | GAGGCGCCTTACTGTTCTTG | S-receptor kinase homolog precursor-like |
| 104 | SEM237 | AGGGAAAGAGACGAGGGAGA | CGTATCTCCGACCACTCCAC | hypothetical protein |
| 105 | SEM238 | CTCTCCCCCAACTCTCTCTG | TCCGACGTCAACGTCTCAG | protein kinase domain containing |
| 106 | SEM239 | CGAGAAACCGTGTCCCCTA | CCCTCTCCCTCTTCCTCCT | CAD1 (constitutively activated cell death 1);oxidoreductase |
| 107 | SEM246 | AATCGATCTTAGGGCCGGTA | ACGCCGACGAGTGAGGAC | hypothetical protein |
| 108 | SEM250 | ACGACTGTTTGTTCGTGCTC | TTCAAAGGGGCTATCTTGCT | flavonol 4'-sulfotransferase |
| 109 | SEM254 | ACCTTACAGAGCCCACTGCT | TCGCGATAATGAGATTGAGC | uv-damaged dna-binding |
| 110 | SEM255 | CGGCGTCCACTGAAAGAG | CAGCCTCGAGTTGGGATG | hypothetical protein OsI_008838 |
| 111 | SEM257 | TGCTGGAGACGGAGTAGCTT | ATCAGGCAAGCACACAATCA | Hit not found |
| 112 | SEM258 | GGAAGAGGAGGCTTCGAGAT | CTGGATAATCACGCCCAAAT | TPA_exp: GRP21 |
| 113 | SEM261 | CCATCCATCCTCTCATCTCC | AAGAGTGCTTGAGCGGATCT | hypothetical protein |
| 114 | SEM263 | AGCCTCTGACGCTAAGATCC | CACACGCTGCAGATGTTGTT | hypothetical protein OsJ_008782 |
| 115 | SEM265 | ACACTAGCTAGCCAGCCACA | GAAGCGAGGCTATGGCTATG | Hit not found |
| 116 | SEM271 | AGCAGATTCACTTCGCCACT | CGATGAGCTTGGAGAGGAG | silverleaf whitefly-induced protein 1 |
| 117 | SEM273 | TTTCTTTTCGTCACACCCAAT | ACTCCCGTCACTCACCTGAC | cdc2 protein kinases-like |
| 118 | SEM275 | TCTCATCGGATTCACACACA | GGGCAGCTTCGTAATGGT | iron deficiency protein Ids3 |
| 119 | SEM276 | AACCCGTTCTTCTTCCCCTA | CAGAGGGAGATTTGCCATGT | indeterminate spikelet 1 |
| 120 | SEM279 | AACCTAAACGACGACGATGG | AGCGAGGAAACGTCGTACAT | phospholipid transfer protein |
| 121 | SEM280 | ATGGAGCTCCGTCTTCTTGT | AGTACCGTAGTTGGGGGTTG | peroxidase atp8a |
| 122 | SEM282 | GAACCTCGCAGTCTTCACAA | CACTACCTGCCTTTCTCTCG | retrotransposon protein, putative, Ty1-copia subclass |
| 123 | SEM285 | TCCTTGAACCTCGCAGTCTT | CTACCTGCCTCTCTCGTTCC | retrotransposon protein, putative, Ty1-copia subclass |
| 124 | SEM287 | CCAATTCAACAAGCATCGAG | GGGAGGACATGAAGTCTGGT | branched-chain amino acid aminotransferase |
| 125 | SEM288 | TCCGATCACAATCACAGACC | GCTGCAGCAGATGACAAACT | ring-h2 finger proteinexpressed |
| 126 | SEM290 | AAACGCAAACCCTTATCTCG | GCTTGGAGGTCACCTTCTTC | Proteasome subunit alpha type 4-1 |
| 127 | SEM294 | CCATACCCTGTACCGTACCC | GATGCTTGCATTCATCCTTG | hypothetical protein OsJ_014087 |
| 128 | SEM295 | CACCTCCCAGACTCTTCTCC | GTGACACCATGGTCCTGAAG | Hit not found |
| 129 | SEM297 | CACCAAACAGACTCGCATTT | CGGATCGAACTCTGTGACAT | g-patch domain containing |
| 130 | SEM298 | ACGCGAGAGGGAGAGAGATA | GTCAGCAGCACGAACAGC | Hit not found |
| 131 | SEM302 | GCGGTTTCTTGTTTTCCTTC | ACCACGACCTCGATCTCAAC | Hit not found |
| 132 | SEM303 | CGAAAACCCTCAAACCCTAA | CTCCTCTAGCTTCCGCTTGT | hypothetical protein |
| 133 | SEM306 | ACCACCAATACCACCACCAC | TCGACGTTGGACTTGAGAAG | ccr4-not transcription complex subunit 7 |
| 134 | SEM307 | CAAACTTTTGCCCGATAGGT | CGGAGCATACCAAGTGAAGA | Hit not found |
| 135 | SEM308 | TCTCGACTCCCCTAATCACC | CGGACAGAAAGATCGCAGTA | Hit not found |
| 136 | SEM310 | AAGAAACCAACCCTCAAAGC | GTAGGGTAGCGCTGGGTAAT | Hit not found |
| 137 | SEM313 | GAGGGAACACATCCCTTCTC | GCCGTAGATGAAGACCTCCT | bicoid-interacting 3 |
| 138 | SEM314 | GAATATAACCGCCACCTTGC | TGGCTTTCCATCTCGTGACT | zinc transporter |
| 139 | SEM315 | GAGGTCCTGGGAGAGACAGA | GTCTGGCCCGTAAGCTGT | membrane protein-like |
| 140 | SEM319 | ATCGTCATCGCAAAATGC | CAACCGGAGGCACTGAGTA | Hit not found |
| 141 | SEM320 | GAGGCAGCTCGACGACAC | GTCAGCTCCGCTCCTGCT | Hit not found |
| 142 | SEM321 | GTCCGTCTCCACTCGAAAAC | GCGGTTGAGGTCGAGGTAG | 6b-interacting protein 1 |
| 143 | SEM327 | CTCCCCTCTCGCTCATCA | AGGTTGACGATGGTGGTGAC | peroxisomal Ca-dependent solute carrier |
| 144 | SEM328 | TCTTGCCTGTTCGTCTTCCT | ATTCCGATTCCGATTCCAAC | Transcriptional corepressor LEUNIG |
| 145 | SEM329 | CACCCAGCTCAAGTACAGCA | GCCTGTAAAAGCCTCCTGTG | beta-1,3-glucanase precursor |
| 146 | SEM332 | CCGCAAGGAAGAACACCTT | GCAGTGGAAGTCGACGTAGG | rna recognition motif-containing |
| 147 | SEM336 | GCCAGGGTTCTTCAAGTGAT | TTCGTCATAGCCATCGTCAT | ubiquitin-conjugating enzyme -like |
| 148 | SEM337 | AGCAATGGTACGCACAAGAG | TTGCTAGTCGTCGTTCTTGG | Hit not found |
| 149 | SEM338 | GATCGGATCGAGAGGAGTTTT | ATACGACGAGGACGAAGTGG | nodulin21 family |
| 150 | SEM339 | AAGCGAGCGTACACCAAATC | ACGGCTCAGATGGTTGAGAG | Hypoxia induced protein conserved region containing protein, |
| 151 | SEM341 | GTGGTTTGAGTACGCTCGTG | AGAGGGATGGCAGTATCCAG | Glucan 1,3-beta-glucosidase precursor |
| 152 | SEM344 | CGTGCGCTCTCTCTCTCTCT | ATTTTGAGATGGCTGCATCA | enhancer of rudimentary |
| 153 | SEM349 | CGAGAGGCCTTCTCTCTCTG | CGCTGACGTAGTCCTGGTAG | symbiosis-related protein-like protein |
| 154 | SEM350 | CCAATGGAGACGACACTCCT | GCGGACGTAGATGGAGAAGA | ring-h2 zinc finger protein |
| 156 | SEM351 | CGACTGTGGGAGGAGTTTGT | TTGCAGCAGTTGCTAGCTGT | Hit not found |
| 157 | SEM353 | TTGCTTCTGTTGGGTTTCAA | TGGTTAAGGTTTGTCGGTGA | reverse transcriptase family member |
| 158 | SEM355 | ACCAAATCCAAACACAGCAG | CGATGGTTGAGAGCTTGTGT | Hypoxia induced protein conserved region containing protein |
| 159 | SEM358 | CTGGCCTCAAGAGGAAACTG | ACCAACCTCTTGACCAGCAC | Hit not found |
| 160 | SEM361 | GTAGCCGTGGAGCATGAAGT | CTGCTGCCATTAGGAGCAAT | Hit not found |
| 161 | SEM366 | CCACCTCTTCTGCCAAGAAC | CATCTTAAACTCCGGTCCACA | Hit not found |
| 162 | SEM367 | AGTCAGCATCCATCCAGTCC | ATTTCTCCTGCCCTCCTCTC | Hit not found |
| 163 | SEM368 | AAACCCTCGCCTCCGATT | CCCAATGGTACCAGCAGAGT | protein tyrosine phosphatase |
| 164 | SEM369 | CGCTTCCATATCTTCTTCTTGG | TGACTCTCCGGTCCCTACAC | ethylene-responsive transcriptional coactivator |
| 165 | SEM371 | GGAGAAGCATTTCAGCAACC | CCCGCTTTTCCTCTTTCTTT | phosphatidate cytidylyltransferase family |
| 166 | SEM372 | GCCAAGCTAAATAGCTGCTG | ACCACCGTTTCTTTCCTGAC | hypothetical protein |
| 167 | SEM373 | GCGACCAAATCTGCCGTAT | CATGTAGTCGAGCGCAGAGA | hypothetical protein OsI_028313 |
| 168 | SEM374 | GCCTCCTCCCTCCTCTTCTA | GACTGGCTCGGAAACCCTA | mitochondrial carrier protein |
| 169 | SEM375 | ATGGAGGCTCGTTGTCTTTG | CCGTAATCGCCTCCACTAAA | ETCHED1 protein |
| 170 | SEM377 | GGAGAGGACGAAACCCTAGC | CGCATTGAACGCAGTTTCTA | wound-responsive protein-related |
| 171 | SEM379 | ACGAGGCCACCATAGAACAT | GCACAAGGTGATTGTGCTGT | hypothetical protein |
| 172 | SEM384 | TAGTAGCAAGCGAGGCGATA | GTCTGTTGCCTTTGATCGTG | Hit not found |
| 173 | SEM390 | GGGGAAGTAAGTCTCAGGTCA | GCCACCACCTCCATTATCTT | cytochrome p450 |
| 174 | SEM391 | GTTCAGACTCGCGTGTTTTT | GCTGAGAACCCTTCAGCTCT | Hit not found |
| 175 | SEM392 | TCATGCTCACCAGCAAAGAC | TCCCGATCAGTGTGTAGACG | hypothetical protein OsJ_000721 |
| 176 | SEM398 | CGTGCAAGCTCCAATATGAT | TGCCACTGTATAGCAGCGTA | Hit not found |
| 177 | SEM400 | CAGCTCATCCTCGTCAACCT | CTCCTCTGCTCCTTGTTGCT | Hit not found |
| 178 | SEM401 | GCTCCATTCATTTCCTCCTC | TTCGATCGATTGATGGTTGA | Alcohol dehydrogenase 2 |
| 179 | SEM403 | CCTGCATCAACCTCTCCAC | GAAGGCGAGAGAGAAGATCG | hypothetical protein |
| 180 | SEM407 | GCGAAACTAGCGCTGCTAAA | GGAGGTTCGGGTACGAGTC | diphosphate-fructose-6-phosphate 1- phosphotransferase |
| 181 | SEM408 | CAGAGCCAGCCAGGTAAAAG | TCATCGTGTGCTGCTGGT | growth-regulating factor 6 |
| 182 | SEM412 | CAAGGCTGCTTCTGGTGTC | CCTCTTTGGGTTCTCTGCTC | Hit not found |
| 183 | SEM415 | CAGCAGACGAGACGAGAGAG | AGGGTGATGAAGGGAATGAG | Rop family GTPase ROP5 |
| 184 | SEM417 | GTCTCCTCCCCCTCCTCTC | AGAAGGAGTCGCTCATCTCG | transcription factor iib |
| 185 | SEM418 | CGGACGTCTCATGTTCTTTG | CAGTGTCCAGTGCAAGTTCC | Hit not found |
| 186 | SEM419 | TGCGTGGTTGATTGAAGAAG | AGAAGCCTCTTCTGCTGCTG | serine threonine protein kinase |
| 187 | SEM421 | CACCCTGCTGGTCTCCTC | TCGACGTCGTGTAGTGAACC | hypothetical protein OsI_010647 |
| 188 | SEM422 | GAAGAGTGGGGACGTCTCAG | GCCAGAGGATGTGGTAGAGG | cytochrome p450 |
| 189 | SEM425 | GTGCCACCAGCAGCAAT | TCTCGTAGCTGCTCGACTTC | Fructose-1,6-bisphosphatase, chloroplast precursor (FBPase) |
| 190 | SEM426 | TCGAGAGCGGTTTCATCTTT | CTTTCCTGTCAGCCAAGTGA | Sugar transporter family protein |
| 191 | SEM427 | AAGTAGCGGAAGCATTAGTTCA | CCAAGTTCCTCCTCACCAGTA | dTDP-glucose 4-6-dehydratase |
| 192 | SEM428 | CAGGAAGAAACAGTAGGAAGCA | AGGTACTTGGCGGTCTTGAT | fructose-bisphosphate aldolase |
| 193 | SEM430 | TCCGACTACCTCAAGTGCAAG | GACGGCATCTTCTTCTTCTCC | sucrose synthase |
| 194 | SEM432 | CGCGTCCGTAGATTAGTAGCTC | AGCGAGTAGATGTTGATGACCC | Sugar transporter family protein |
| 195 | SEM433 | GACACGCCCAAAGGAAAAG | GAGATCCGGACACACATGG | diphosphate-fructose-6-phosphate 1-phosphotransferase |
| 196 | SEM434 | TTCTTGCTTCTTTCTTTCCGTC | TCAAATCGTGCTTGCTTGAG | disease resistance protein (TIR-NBS-LRR class) |
| 197 | SEM435 | AGGCTGAGAGAGCAAAGAAAGA | CCTAGGATCCTTCGGGTTTC | stress-induced protein sti1 |
| 198 | SEM436 | GGTCCCATACATAACACAAGCA | TGCATGAAGAAGCTCAGGTG | disease resistance response protein-related/dirigent protein-related |
| 199 | SEM437 | CCTGGTTCCTGCACTTGTCT | CATCACTTGCCATCTGCATT | NBS-LRR disease resistance protein homologue |
| 200 | SEM439 | CGTCAAGCTGTAGTCCGAGAG | CTCGTCCCAGACCAGGAG | Pathogenesis-related protein PR-1 |
| 201 | SEM440 | AGCAACCTAATCACAGCAACAA | CCATCATCCGATCATCCTTC | disease resistance-responsive family protein |
| 202 | SEM442 | CATTTATTTGCCACCTAGAAGGG | AAACAGAAACCGGACAGCAC | disease resistance protein |
| 203 | SEM443 | GGAATGGGAACAGCCACTAAC | AAGAAGGCTATCGAGGTGGG | dehydration responsive element binding protein |
| 204 | SEM444 | CACGGTTCTCCTGCTGAAAG | GACGGGGTTGTTGAAGGTG | Dehydration-responsive element-binding protein 2D (DREB2D protein) |
| 205 | SEM446 | GAGCAGTCCCTTGCCATGT | GCCGTCGAGTACACCGTC | cold shock protein-1 |
| 206 | SEM447 | TGAGTTCAGTTCCTTCCCC | AGAACTCCAAGGAGCAGCAG | low temperature and salt responsive protein-like |
| 207 | SEM449 | TGGTGTGAGTTAGTGCCTGAGT | TAGAAGGTGTTGATGATGAGCG | Heat shock protein 81-1 |
| 208 | SEM450 | TTCTTGCTTCTTTCTTTCCGTC | AGATGAACACATAGTTGCACCG | light-dependent short hypocotyl 1 |
| 209 | SEM453 | AGCGACATGAGCTACCGTCT | TAGTACCGCGACAGACCTTTCT | cold induced protein-like |
| 210 | SEM454 | GTAACTAGCAGCAACCCTAGCC | ATCCTCTTTTGCCTCCCCT | water-stress protein-like protein |
| 211 | SEM456 | TCGTCCTACAACCACGACTACA | GAGAGGCAAGCAAGGAAAGAT | Saccharum hybrid cultivar soluble acid invertase (ShinvA) mRNA |
| 212 | SEST3 | CCCCGAAGATCAAGGATAGG | CGCATCTCAAATGGGAAAAT | disease resistance protein I2 |
| 213 | SEST4 | CAGGCACTGATGTCATGGAT | GAACTACACTCGCCGCTCAC | early-responsive to dehydration protein |

**Supplementary Table 2:** List of 70 genotypes of sugarcane along with their check lines used for field screening

| **S.No** | **Genotypes** | **Cross lines (Labelled)** |
| --- | --- | --- |
| 1 | CoC671 x BO155 | Co21126 (G1)  Co21127 (G2)  Co21128 (G3)  Co21129 (G4)  Co21130 (G5) |
| 2 | CoSe92423 x CoA0015 | Co21001 (G6)  Co21009 (G7)  Co21025 (G8)  Co21032 (G9)  Co21033 (G10) |
| 3 | BO102GC | Co21074 (G11)  Co21075 (G12)  Co21082 (G13)  Co21083 (G14)  Co21084 (G15) |
| 4 | BO137GC | Co21097 (G16)  Co21100 (G17)  Co21101 (G18)  Co21102 (G19)  Co21108 (G20) |
| 5 | Co671 x CoSe92423 | Co21297 (G21)  Co21299 (G22)  Co21300 (G23)  Co21303 (G24)  Co21305 (G25) |
| 6 | BO92 x NCo310 | Co21335 (G26)  Co21336 (G27)  Co21337 (G28)  Co21338 (G29)  Co21339 (G30) |
| 7 | Co0238 x BO130 | Co21347 (G31)  Co21348 (G32)  Co21349 (G33)  Co21346 (G34)  Co21344 (G35) |
| 8 | BO91 x Co775 | Co21372 (G36)  Co21373 (G37)  Co21374 (G38)  Co21375 (G39)  Co21376 (G40) |
| 9 | CoV14061 x CoSe92423 | Co21400 (G41)  Co21403 (G42)  Co21407 (G43)  Co21408 (G44)  Co21410 (G45) |
| 10 | CoP06436 x Co62198 | Co21494 (G46)  Co21495 (G47)  Co21496 (G48)  Co21498 (G49)  Co21499 (G50) |
| 11 | Co0238 x BO155 | Co21595 (G51)  Co21598 (G52)  Co21593 (G53)  Co21599 (G54)  Co21600 (G55) |
| 12 | BO92 x Co775 | Co21629 (G56)  Co21627 (G57)  Co21626 (G58)  Co21625 (G59)  Co21637 (G60) |
| 13 | BO154 x Co62198 | Co21136 (G61)  Co21137 (G62)  Co21138 (G63)  Co21142 (G64)  Co21143 (G65) |
| 14 | BO139GC | Co21245 (G66)  Co21246 (G67)  Co21251 (G68)  Co21254 (G69)  Co21256 (G70) |
| Check | CoP16437 | |
|  | CoP2061 | |

Where GC= general cross (Female parent known and male parents are unknown)

**Supplementary Table 3:** List of 30 EST-SSR primers selected for association study

| **S.No** | **PRIMERS NAME** | **FORWARD PRIMER** | **REVERSE PRIMER** |
| --- | --- | --- | --- |
| 1 | SEM2 | ACACCGAGCTGTCCCAAT | GCATCTGATGAGCCTGTGAA |
| 2 | SEM58 | CCAACCAACCTCGACATTCT | CCATGTGATCTGACCTGGTG |
| 3 | SEM92 | TGTGCCGTTGCCTAATAACA | GGCAAGCTTCCTCAGTTTCTT |
| 4 | SEM112 | CCTTCTGCAGACGAGTTGAA | ACCTGACCAGCAAATCAACA |
| 5 | SEM117 | GCGTGGCACTGACTACAAGT | CAATGTTCTGTGGTCTGCACT |
| 6 | SEM159 | CTGGTGGAATAACTCGCTGA | CTCAAGGCAAGAACGAATCC |
| 7 | SEM168 | AACGTCAGCCGCTACAACTT | CTTCCCTTTTGCGAAGAAAA |
| 8 | SEM191 | CCAGTCGCGATTCTTCCAC | AAGGGACGGGGAGAAAAATA |
| 9 | SEM199 | CTCTCGAGGAGGTGGATGAG | CTGCAAGTTTGTTGGCTGAA |
| 10 | SEM203 | GCGGCCTCATACGTGTAGAT | TCTCTTCCCCTCACCAGAAA |
| 11 | SEM229 | AGAACCACAACCACCAGGAG | ACAGTTGAATAGGCCGGATG |
| 12 | SEM358 | CTGGCCTCAAGAGGAAACTG | ACCAACCTCTTGACCAGCAC |
| 13 | SEM368 | AAACCCTCGCCTCCGATT | CCCAATGGTACCAGCAGAGT |
| 14 | SEM369 | CGCTTCCATATCTTCTTCTTGG | TGACTCTCCGGTCCCTACAC |
| 15 | SEM375 | ATGGAGGCTCGTTGTCTTTG | CCGTAATCGCCTCCACTAAA |
| 16 | SEM407 | GCGAAACTAGCGCTGCTAAA | GGAGGTTCGGGTACGAGTC |
| 17 | SEM425 | GTGCCACCAGCAGCAAT | TCTCGTAGCTGCTCGACTTC |
| 18 | SEM426 | TCGAGAGCGGTTTCATCTTT | CTTTCCTGTCAGCCAAGTGA |
| 19 | SEM428 | CAGGAAGAAACAGTAGGAAGCA | AGGTACTTGGCGGTCTTGAT |
| 20 | SEM430 | TCCGACTACCTCAAGTGCAAG | GACGGCATCTTCTTCTTCTCC |
| 21 | SEM432 | CGCGTCCGTAGATTAGTAGCTC | AGCGAGTAGATGTTGATGACCC |
| 22 | SEM433 | GACACGCCCAAAGGAAAAG | GAGATCCGGACACACATGG |
| 23 | SEM434 | TTCTTGCTTCTTTCTTTCCGTC | TCAAATCGTGCTTGCTTGAG |
| 24 | SEM435 | AGGCTGAGAGAGCAAAGAAAGA | CCTAGGATCCTTCGGGTTTC |
| 25 | SEM436 | GGTCCCATACATAACACAAGCA | TGCATGAAGAAGCTCAGGTG |
| 26 | SEM437 | CCTGGTTCCTGCACTTGTCT | CATCACTTGCCATCTGCATT |
| 27 | SEM439 | CGTCAAGCTGTAGTCCGAGAG | CTCGTCCCAGACCAGGAG |
| 28 | SEM440 | AGCAACCTAATCACAGCAACAA | CCATCATCCGATCATCCTTC |
| 29 | SEM454 | GTAACTAGCAGCAACCCTAGCC | ATCCTCTTTTGCCTCCCCT |
| 30 | SEM456 | TCGTCCTACAACCACGACTACA | GAGAGGCAAGCAAGGAAAGAT |


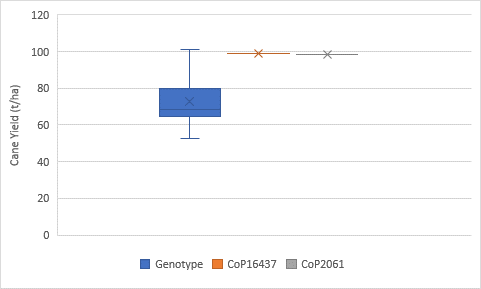


**Supplementary Figure: 1** Distribution pattern of the yield of 70 sugarcane genotypes with two check lines.

**
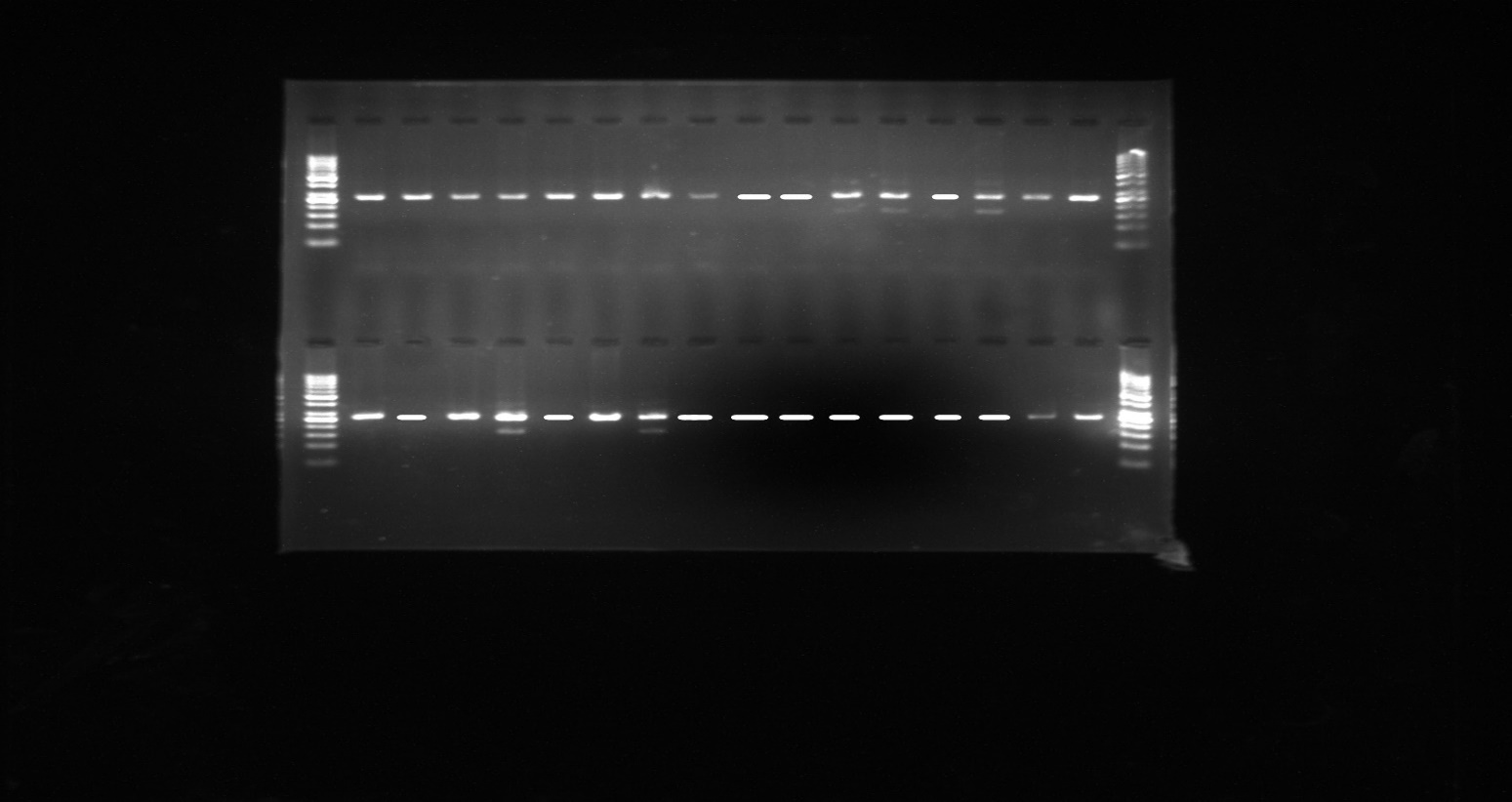
**

**M 1 2 3 4 5 6 7 8 9 10 11 12 13 14 15 16 17**

**M 18 19 20 21 22 23 24 25 26 27 28 29 30 31 32 33 34 M**

250bp

200bp

150bp

100bp

50bp


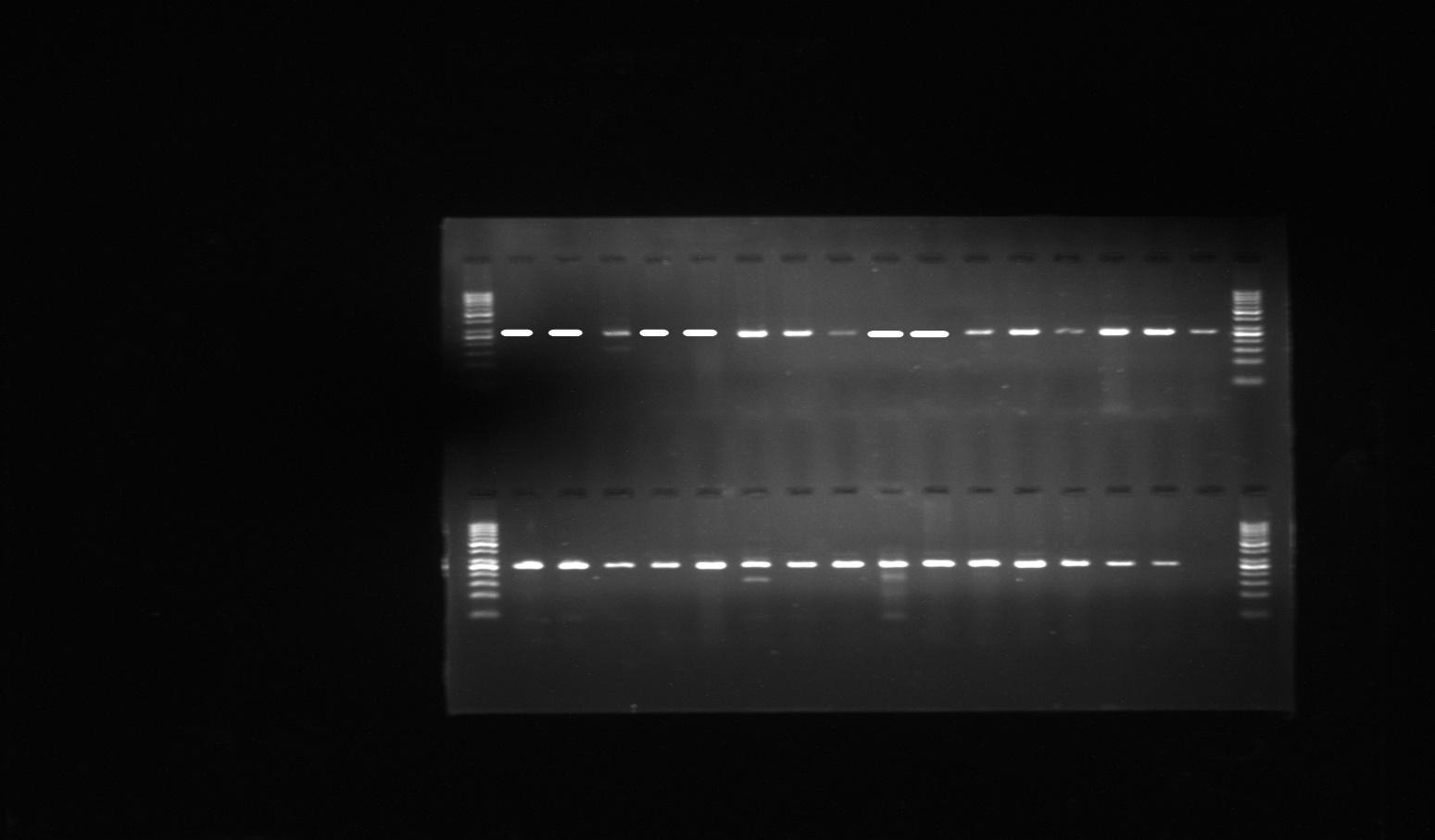


M 35 36 37 38 39 40 41 42 43 44 45 46 47 48 49 50 51M

M 52 53 54 55 56 57 58 59 60 61 62 63 64 65 66 67 68 69 70 M

**250bp**

**200bp**

**150bp**

**100bp**

**50bp**

**Supplementary Figure 2:** EST-SSR profiles generated by SEM435 primers on 70 accessions of sugarcane. 1-70 refers to the accession number and M refers to the DNA ladder of 50bp.
